# Evaluation of Steroid-Induced Osteoporosis Prevention Using Tracing Reports in Collaboration between Hospitals and Community Pharmacists

**DOI:** 10.3390/pharmacy12030080

**Published:** 2024-05-15

**Authors:** Nonoko Ishihara, Shuji Yamashita, Shizuno Seiki, Keito Tsutsui, Hiroko Kato-Hayashi, Shuji Sakurai, Kyoko Niwa, Takuyoshi Kawai, Junko Kai, Akio Suzuki, Hideki Hayashi

**Affiliations:** 1 Laboratory of Home Team Care Pharmacy, Gifu Pharmaceutical University, 1-25-4 Daigaku Nishi, Gifu 501-1196, Gifu, Japan; 185004@gifu-pu.ac.jp (N.I.); kai@uno-upd.co.jp (J.K.); 2 Laboratory of Community Pharmaceutical Practice and Science, Gifu Pharmaceutical University, 1-25-4 Daigaku Nishi, Gifu 501-1196, Gifu, Japan; syamashita@gifu-pu.ac.jp (S.Y.); shizuno9641@gmail.com (S.S.); 195074@gifu-pu.ac.jp (K.T.); 3 Department of Pharmacy, Gifu University Hospital, 1-1 Yanagido, Gifu 501-1194, Gifu, Japan; kato.hiroko.m7@f.gifu-u.ac.jp (H.K.-H.); sakurai.shuji.z6@f.gifu-u.ac.jp (S.S.); niwa.kyoko.a4@f.gifu-u.ac.jp (K.N.); suzuki.akio.k5@f.gifu-u.ac.jp (A.S.); 4 Laboratory of Community Healthcare Pharmacy, Gifu Pharmaceutical University, 1-25-4 Daigaku Nishi, Gifu 501-1196, Gifu, Japan; kawai-ta@gifu-pu.ac.jp; 5 Laboratory of Advanced Medical Pharmacy, Gifu Pharmaceutical University, 1-25-4 Daigaku Nishi, Gifu 501-1196, Gifu, Japan

**Keywords:** glucocorticoid-induced osteoporosis, evidence–practice gap, GIOP guidelines, tracing reports, pharmacist

## Abstract

Glucocorticoid-induced osteoporosis (GIOP) is a side effect of glucocorticoid (GC) treatment; however, despite established prevention guidelines in various countries, a gap persists between these guidelines and clinical practice. To address this gap, we implemented a collaborative intervention between hospitals and community pharmacists, aiming to assess its effectiveness. Pharmacists recommended to the prescribing doctor osteoporosis treatment for patients who did not undergo osteoporosis treatment with a fracture risk score of ≥3 via tracing reports (TRs), between 15 December 2021, and 21 January 2022. Data were extracted from electronic medical records, including prescriptions, concomitant medications, reasons for not pursuing osteoporosis treatment, and TR contents. Of 391 evaluated patients, 45 were eligible for TRs, with 34 (75.6%) being males. Prednisolone was the most common GCs administered, and urology was the predominant treatment department. Among the 45 patients who received TRs, prescription suggestions were accepted for 19 (42.2%). After undertaking the intervention, guideline adherence significantly increased from 87% to 92.5%. This improvement indicates that TRs effectively bridged the evidence–practice gap in GIOP prevention among GC patients, suggesting their potential utility. Expansion of this initiative is warranted to further prevent GIOP.

## 1. Introduction

Glucocorticoid-induced osteoporosis (GIOP) is a serious iatrogenic adverse event caused by long-term glucocorticoid (GC) treatment. GIOP can cause fractures even when bone mineral density (BMD) is maintained [1]. Fractures may decrease the patient’s activities of daily living and quality of life [2,3] and increase the burden on the family and others. Thus, prevention of GIOP as early as possible after the start of GC treatment is important [4,5].

Efforts have been made, including interventions by pharmacists who utilize educational programs for both general practitioners and patients [6,7], monitoring of doses for GC and osteoporosis drugs [8], and prescribing suggestions based on feedback from pharmacists to physicians [9]. In addition, academic societies in various countries have established guidelines aimed at promoting appropriate management of GIOP [10,11,12,13,14].

In Japan, the “Guidelines for the Management and Treatment of Steroid-induced Osteoporosis 2014 Revised Edition” was formulated to guide the appropriate management of GIOP [15]. The GIOP guidelines incorporated a simple evaluation method using fracture risk scores and the pharmacotherapy recommend for GIOP (alendronate and risedronate as first-line treatment, with teriparatide, ibandronate, alfacalcidol and calcitriol as alternative treatments). Several studies evaluated treatment compliance with the GIOP guidelines [16,17]. We conducted a factual investigation on the GIOP guideline adherence rate of long-term outpatient GC patients at a community pharmacy. Although the GIOP guidelines compliance rate was high compared to previous reports, some patients did not receive GIOP prophylaxis despite the GIOP guideline recommendations for pharmacotherapy [18]. The percentage of patients who received recommended preventive care in various areas, not just GIOP, was 54.9% [19], indicating that a problematic gap exists between evidence and actual practice. Furthermore, the average guideline adherence rate is 67%, with wide variation among doctors and guidelines [20]. For this reason, we attempted to close the evidence–practice gap in GIOP prophylaxis via collaboration between hospitals and community pharmacists. Gifu University Hospital Pharmacy Department (hospital pharmacy) and the community pharmacies attend regular joint training sessions as part of a collaborative effort between hospitals and community pharmacists. In the current GIOP prevention intervention, tracing reports were used to propose prescriptions. Tracing reports, which are also referred to as medication information documents, are information documents that provide nonurgent information from community pharmacists to physicians, such as adherence and concomitant medication status of patients [21].

Appropriate pharmaceutical management through collaboration between hospitals and community pharmacists has been useful in outpatient cancer chemotherapy, chronic kidney disease, and residual medication reconciliation [22,23,24]. Residuals are generally caused by patients forgetting to take prescribed their medications or taking them incorrectly, and reconciliation such as reducing the number of prescription days is often performed at community pharmacies. However, to our knowledge, prescribing suggestions for GIOP prevention have not been implemented. The purpose of this study was to evaluate the usefulness of tracing reports to facilitate communication between hospitals and community pharmacists. The implementation status of prescribing suggestions for osteoporosis treatment using tracing reports and the GIOP guideline adherence rate collected from electronic medical records and other sources at Gifu University Hospital were retrospectively assessed.

## 2. Materials and Methods

### 2.1. Overview of Intervention

**Figure 1:**
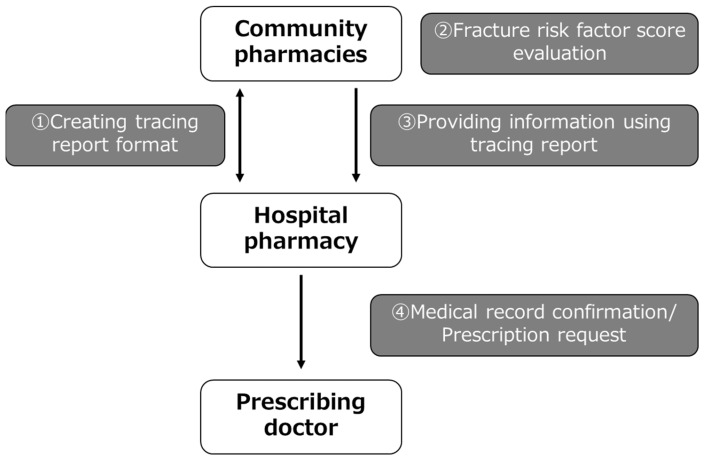
Outline of the intervention showing the collaboration between hospitals and community pharmacists.

An overview of the intervention is shown in Figure 1. Discussions were held between the hospital pharmacy and community pharmacies, and tracing reports with a unified format were created for proposing osteoporosis treatment prescriptions. Based on the GIOP guidelines, a table was created for the tracing reports in which pharmacists can enter each fracture risk factor score based on the patient’s condition, making the eligibility for osteoporosis prescription recommendations easier to determine. In addition, the first-line osteoporosis treatments are listed based on the GIOP guidelines. The GIOP guidelines provide general guidance to patients who use or plan to use oral GCs for more than 3 months. Each fracture risk factor is evaluated, including the presence of existing fracture, age, steroid dosage, and BMD. Although there is a trend to adopt FRAX for fracture risk assessment of GIOP in patients receiving long-term GCs, each risk factor extracted from a unique cohort is weighted and scored, and the risk is assessed by the total score in Japan. If the total score is 3 or higher, drug therapy is recommended [15]. For this intervention, three of the four fracture risk factors in the GIOP guidelines (presence of preexisting fracture, age, and GC dosage) were used by the community pharmacies to evaluate fracture risk factor scores. Community pharmacists conducted GIOP risk assessments in those patients prescribed long-term GCs and recommended prophylactic treatment for those who were not prescribed an osteoporosis treatment according to a standardized format when the total score was 3 or higher. These recommendations were communicated to hospital pharmacists via the tracing reports. The hospital pharmacists verified the community pharmacist’s recommendation using the electronic medical record and ultimately communicated the recommendation to initiate prophylactic therapy to the prescribing doctor using the electronic medical record system. In addition, community pharmacies provided information to the hospital pharmacy about the number of patients with a total score of less than 3 and the number of patients with a total score of 3 or higher who were prescribed an osteoporosis treatment. 

### 2.2. Patients Identified and Intervention

The survey period for this study was from 15 December 2021 to 21 January 2022. Patients who were prescribed GCs for more than 3 months at Gifu University Hospital and the GC was dispensed at Gifu Pharmaceutical University Pharmacy, V Drug Pharmacy (Gifu University Hospital Store), Tanpopo Yakkyoku Gidaimaeten, Kirari Pharmacy, Ain Pharmacy Gidaibyouinmae, or Nihon Chouzai Gidaimae Pharmacy were enrolled in the study. Patients younger than 18 years, patients using GCs on an irregular basis, and patients using GCs as supportive care with outpatient cancer chemotherapy were excluded. Patients were identified whether eligible or not during the dispensing process when they visited participating community pharmacies. All patients who brought a prescription containing GCs during the intervention period and who were confirmed to have been taking GCs for more than 3 months were included, regardless of whether it was their first time taking GCs or whether they had visited the pharmacy before. However, only the first visit during the intervention period was included. Also, information on each fracture risk factor (age, GC dose, history of fracture), eligibility for exclusion criteria, and whether or not osteoporosis treatments were prescribed was obtained from prescriptions and electronic drug histories. However, if it was unclear, it was confirmed during an in-person consultation at the community pharmacy. Based on the information obtained, fracture risk was assessed and a tracing report was completed. Pharmacists at community pharmacies who participated in the intervention received an online explanation about the intervention from the authors during a joint training session with hospital pharmacy departments. The explanation included the significance and outline of the intervention, GIOP guidelines, how to write a standardized tracing report, and how to identify target patients. The intervention procedure was simple and did not require any special training to participate.

### 2.3. Evaluation of the Usefulness of This Intervention

For patients with a fracture risk score of 3 or higher and no osteoporosis treatment prescription (patients for whom prescriptions were proposed in tracing reports), the following information was retrospectively extracted from the electronic medical records of Gifu University Hospital: presence or absence of existing fractures, age, GC dosage, GC type, gender, medical department, non-GC prescription (concomitant medications), whether or not an osteoporosis treatment was added, the reason an osteoporosis treatment was not added, and the contents of tracing reports. The number of patients with a total score of less than 3 and the number of patients with a total score of 3 or more who were prescribed an osteoporosis treatment were determined based on reports from each pharmacy. A score of 0 was defined as no or unknown preexisting fracture in the score assessment. The GIOP guideline adherence rate was defined as the number of patients with a fracture risk score of 3 or higher who received drug therapy/number of patients with a fracture risk score of 3 or higher. The intervention as a collaborative effort between hospitals and community pharmacists and this study was initiated and evaluated, respectively, from 15 December 2021 to 21 January 2022. Of the eligible patients, those who met the exclusion criteria were excluded from the scoring at the community pharmacy. Therefore, only data from patients who were scored were included.

### 2.4. Statistical Analysis

Changes in GIOP guideline adherence rates before and after this intervention were examined using a McNemar test. The acceptance rates of osteoporosis treatment proposals among patients with a total score of 3 or higher and no osteoporosis treatment prescription were compared with the Fisher’s exact test. *p* < 0.05 was considered a significant difference. Statistical analyses were conducted using EZR, which expands the functions of R and R Commander. EZR is provided free of charge on the website of the Department of Hematology, Saitama Medical Center, Jichi Medical University [25].

### 2.5. Ethical Considerations

This study was conducted in compliance with the Ethical Guidelines for Medical Research Involving Human Subjects and was approved by the Ethical Review Committee of Gifu Pharmaceutical University (5-1, Approved on 27 November 2023) and the Ethical Review Committee for Medical Research and Other Research, Graduate School of Medicine, Gifu University (2022-019, Approved on 13 October 2023).

## 3. Results

### 3.1. Study Population

**Figure 2:**
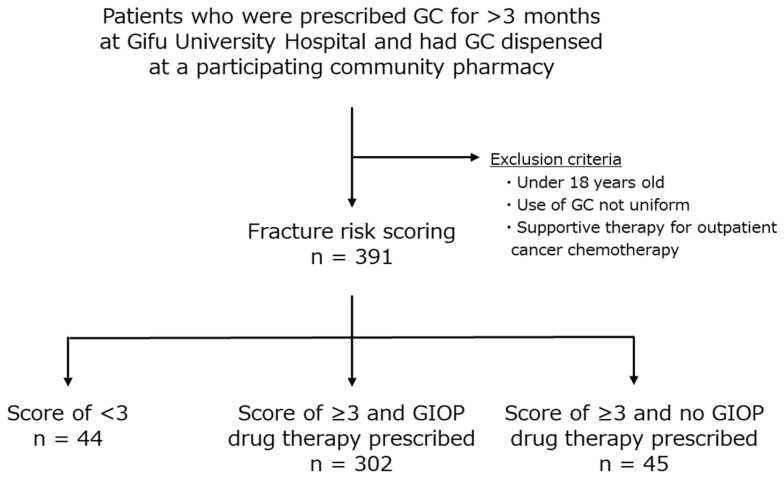
Tracing report target patient selection flowchart. GC: glucocorticoid, GIOP: glucocorticoid-induced osteoporosis.

Of the 391 patients who were scored, 44 patients had a score of less than 3, 302 patients had a score of 3 or more and were prescribed an osteoporosis treatment, and 45 patients had a score of 3 or more and were not prescribed an osteoporosis treatment. Thus, 45 patients were eligible for the proposed osteoporosis treatment by sending tracing reports. Also the number of patients who met the exclusion criteria was unknown (Figure 2). Of these 45 patients, 34 (75.6%) were males and 11 (24.4%) were females. The patients were treated in the following departments: urology (n = 19, 42.2%), hematology and infectious diseases (n = 7, 15.6%), and general internal medicine (n = 5, 11.1%). The most frequently taken GCs were prednisolone (PSL) and methylprednisolone, in that order, with a mean dose of 5.9 ± 4.6 mg/day PSL equivalent (Table 1).

### 3.2. Evaluation of Fracture Risk Factor Scores

Table 2 shows the fracture risk scores for the 45 patients who were sent tracing reports. Thirty-nine patients (86.7%) had no or unknown preexisting fractures (score 0), and six patients (13.3%) had preexisting fractures (score 7). Thirty-two patients (71.1%) were 65 years or older (score 4), followed by patients between 50 and 65 years (score 2) (n = 8, 17.8%) and patients under 50 years (score 0) (n = 5, 11.1%). The largest number of patients received 5–7.5 mg PSL equivalent (score 1) (n = 24, 53.3%), followed by 7.5 mg or more (n = 11, 24.4%) and 5 mg or less (n = 10, 22.2%). Sixteen respondents (35.6%) had a score of 5, nine respondents (20.0%) had a score of 4, and eight respondents (17.8%) had a score of 8.

### 3.3. Change in GIOP Guideline Adherence Rate Due to This Intervention

Of the 45 patients sent tracing reports in this intervention, osteoporosis treatment prescriptions were added to 19 patients (42.2%). The drug classes added included bisphosphonates in 12 cases and active vitamin D_3_ in 7 cases. The GIOP guideline adherence rate increased significantly from an 87.0% adherence rate before this intervention to a 92.5% adherence rate after this intervention (Table 3). In addition, the osteoporosis treatment acceptance rates were 44.1% for males and 36.4% for females and were not statistically significant. No differences in the acceptance rates of prescription suggestions according to age, GC dosage, or presence of preexisting fractures were detected (Table 4).

### 3.4. Classification of Reasons for no Additional Osteoporosis Treatment

**Figure 3:**
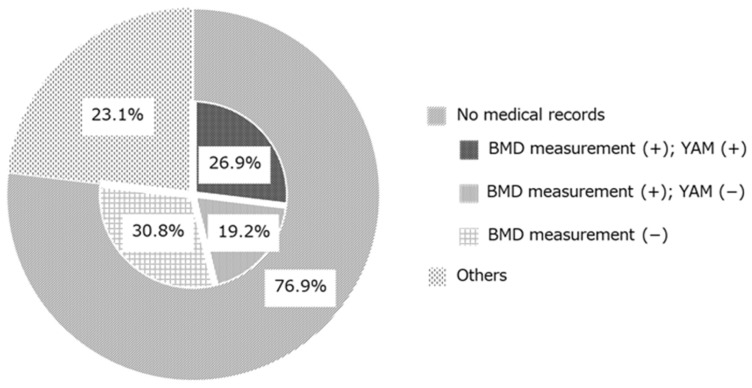
Classification of reasons why osteoporosis treatments were not prescribed after the prescription proposal (n = 26). BMD: bone mineral density; YAM: young adult mean.

The reasons for no additional osteoporosis treatment are shown in Figure 3. Although drug therapy was recommended based on the GIOP guidelines and a prescription was proposed by the tracing reports, the treatment recommendation was not accepted in 26 cases. In 20 cases (76.9%), the reason for not adding prescription was not clearly documented in the electronic medical records. Therefore, these reasons were classified according to whether BMD measurements and YAM values were recorded. In 7 cases (26.9%), patients underwent bone densitometry testing and the Young Adult Mean (YAM) value was stated. In 5 cases (19.2%), patients underwent bone densitometry testing but the YAM value was not stated. In 8 cases (30.8%), BMD was not measured. Other cases (n = 6; 23.1%) included the patient’s intention, undergoing dental treatment, patient relocation, scheduled bone densitometry, termination of GC administration after sending the tracing reports, and death after sending the tracing reports. Patient’s intention means that the patient did not accept osteoporosis treatment despite being recommended by the doctor after receiving the proposal from the pharmacist.

## 4. Discussion

This is the first ever study to assess the effectiveness of a collaborative intervention between hospitals and community pharmacists aimed at preventing GIOP to our knowledge. The key tool facilitating their collaboration was uniformly formatted tracing reports based on GIOP guidelines, which were developed jointly by both sets of pharmacists. Although GIOP guidelines remain incompletely harmonized internationally, with content varying by country, interventions based on these guidelines have been undertaken in various regions [26,27]. Despite reported enhancements in prevention interventions for GIOP, a treatment gap persists, necessitating effective interventions to address it [28,29].

As far as we know, the GIOP guidelines do not have clear diagnostic criteria but often include criteria for starting treatment [10,11,12,13,14]. For this reason, the basis for calculating the number of patients with GIOP is unclear, and there is a general tendency to use patients who have been taking GCs for a long time as a substitute number. Approximately 0.79% to 1.2% of adults suffer from osteoporosis due to long-term use of GCs [30,31], and there is a report that 30–50% of patients taking long-term GC experience fractures [32]. Also, according to a network meta-analysis by Deng et al., alendronate and risedronate reduced the risk of vertebral fracture by 52% and 50%, respectively, in patients taking long-term GCs [33]. The GIOP guideline adherence rates significantly improved from 87.0% before the intervention to 92.5% after implementation of the intervention. Improvements in the GIOP guideline adherence rates in this study mean that additional osteoporosis treatment could be started in patients with GIOP. Therefore, in this study, since alendronate (n = 7) and risedronate (n = 3) were added after pharmacist intervention, it could be estimated that it might theoretically be possible to prevent 1–2 fractures. Based on the above, the improvement in the GIOP guideline adherence rates in this study was considered to have clinical significance. The evidence–practice gap for GC patients improved, suggesting that this intervention was useful and pharmacists can contribute to GIOP prevention. In this intervention, if a prescription proposal was not accepted, the reason was confirmed in the electronic medical record. As a result, the majority of cases (76.9%) did not have a clearly stated reason, although the bone densitometry testing and YAM values were recorded (Figure 3). When YAM values were listed, 83.3% (5/6 cases) exceeded the YAM value that would give a score of 0 according to the GIOP guidelines. The 2004 edition of the “Guidelines for the Management and Treatment of Steroid-induced Osteoporosis” recommended an algorithm to perform bone densitometry testing. If the PSL equivalent was less than 5 mg/day, general guidance and follow-up were recommended [34]. Thus, when BMD was measured, the BMD measurement may have been sufficient and no additional osteoporosis treatment was prescribed in some cases. As mentioned above, fractures can occur in GIOP even if BMD is maintained [1]. Thus, the newer GIOP guidelines recommend osteoporosis treatment when the total fracture risk factor score is 3 or higher [15]. For this reason, prescriptions for osteoporosis treatment may be necessary in addition to providing information on the contents of the GIOP guidelines.

More than half of the patients in the study were 65 years or older (score 4), and, even when age was the only assessment, a score of 3 or higher was attained and drug therapy was recommended in many cases. This may be because elderly patients have multiple diseases and are more likely to visit multiple departments and take multiple medications.

The male-to-female ratio of patients sent tracing reports in this study was approximately 3:1 (34 males, 11 females) (Table 1). Although the total number of patients taking GCs long term in this study is unknown, 47.1% of patients taking GCs long term were males in a previous study at the same medical institution [18]. Thus, gender bias among patients taking GCs long term in this study is unlikely given the patient background. Hence, more male patients taking GCs long term may be overlooked for GIOP prophylaxis compared to female patients. The male-to-female ratio of patients taking long-term GCs is approximately 1:1 in Japan [35], and no significant differences in fracture risk based on gender were detected in overseas patients [36]. However, the prevalence of osteoporosis is generally higher in women [37]. The perception may be that GIOP, which is secondary osteoporosis, is more likely to develop in women. Thus, increasing awareness of the gender of patients taking GCs long term and paying attention to male patients may contribute to GIOP prevention.

As part of the collaborative intervention, hospitals and community pharmacies exchanged opinions and developed a unified tracing report form for osteoporosis treatment prescribing suggestions based on the GIOP guidelines. The tracing reports were useful for improving the consistency and efficiency of care [38]. In this intervention, the utilization of tracing reports with a unified format may have lowered the psychological hurdles faced by pharmacists when making prescription proposals to prescribing doctors and may have reduced the workload associated with creating tracing reports. As a result, implementing prescribing suggestions more proactively may improve GIOP guideline adherence rates.

This study has several limitations. First, data were extracted based on information provided by tracing reports from community pharmacies that filled prescriptions issued by Gifu University Hospital. Because of this, the analysis was specific to prescriptions from university hospitals. Thus, the results may not be generalizable. The scale of the survey should be expanded to include community pharmacies that accept prescriptions from other medical facilities, such as clinics and community hospitals. Second, as this intervention utilized modified GIOP guidelines, recommendations to start drug therapy may have been overlooked in some patients. The evidence–practice gap may be closed further by recommending bone densitometry testing for the appropriate evaluation of fracture risk based on the GIOP guidelines. Third, although a significant improvement in the GIOP guideline adherence rate was observed, the usefulness of the drug was not evaluated by following patients after administration and confirming the presence or absence of actual fractures. The study’s primary endpoint, GIOP guideline adherence rate, was a surrogate outcome. A prospective cohort study with actual fractures as the end point to evaluate the usefulness of prescription suggestions for GIOP prevention based on GIOP guidelines by pharmacists should be conducted. One other potential limitation was that the study did not investigate harms of treating GIOP when care was delivered according to guidelines. This study showed that guideline-concordant care prompted the need for further BMD testing, which has potential economic consequences to the health system.

## 5. Conclusions

In this study, an intervention to prevent GIOP by utilizing uniform tracing report forms developed based on the GIOP guidelines was evaluated. Although this study had several limitations, including limited generalizability, adherence to the GIOP guidelines improved through this intervention, suggesting that proposing osteoporosis treatment prescriptions using tracing reports may be useful. Activities to raise awareness of GIOP prevention in conjunction with wider implementation of this intervention may reduce the risk of fractures in patients receiving long-term GC treatment.

## Figures and Tables

**Table 1:** Background of patients eligible for tracing report sending (n = 45).

Patient Characteristics	n	Frequency (%)
Gender		
male	34	(75.6%)
female	11	(24.4%)
Age group		
<50 years	5	(11.1%)
50–65 years	8	(17.8%)
≥65 years	32	(71.1%)
Clinical department		
Urology	19	(42.2%)
Hematology and infectious diseases	7	(15.6%)
General internal medicine	5	(11.1%)
Gastroenterology	4	(8.9%)
Dermatology	3	(6.7%)
Immunology/Endocrinology	3	(6.7%)
Respiratory medicine	3	(6.7%)
Nephrology	1	(2.2%)
GC ^a^ type		
Prednisolone	29	(64.4%)
Methylprednisolone	13	(28.9%)
Hydrocortisone	2	(4.4%)
Betamethasone	1	(2.2%)
GC dose, mg/day (PSL equivalent)	5 (1–30) ^b^	

^a^ Multiple prescriptions were possible; ^b^ Median (range); GC: glucocorticoid, PSL: prednisolone.

**Table 2:** Fracture risk factor score evaluation (n = 45).

Fracture Risk Factor			Score	n	Frequency (%)
History of fracture					
yes			7	6	(13.3%)
no or unknown			0	39	(86.7%)
Age group					
<50 years			0	5	(11.1%)
50–65 years			2	8	(17.8%)
≥65 years			4	32	(71.1%)
GC dose category (PSL equivalent in mg/day)					
<5 mg/day			0	10	(22.2%)
5–7.5 mg/day			1	24	(53.3%)
≥7.5 mg/day or more			4	11	(24.4%)
Total score					
			3	6	(13.3%)
			4	9	(20.0%)
			5	16	(35.6%)
			6	1	(2.2%)
			7	2	(4.4%)
			8	8	(17.8%)
			10	1	(2.2%)
			11	1	(2.2%)
			15	1	(2.2%)

GC: glucocorticoid, PSL: prednisolone.

**Table 3:** Changes in guideline adherence rate (n = 347).

	Guideline Adherence		Adherence Rate ^a^ (%)	McNemar Test
	Yes	No		
Before the intervention	302	45	87.0	*p* < 0.001
After the intervention	321	26	92.5	

^a^ Number of patients with a total score of 3 or higher and receiving drug therapy/Number of patients with a total score of 3 or higher.

**Table 4:** Prescription proposal acceptance rate by factor (n = 45).

	Prescription Acceptance		Prescription Acceptance Rate (%)	Fisher’s Exact Test
	Yes	No		
Gender				
male	15	19	44.1	*p* = 0.736
female	4	7	36.4	
Age group				
<50 years	2	3	40.0	*p* = 0.645
50–65 years	2	6	25.0	
≥65 years	15	17	46.9	
GC dose category (PSL equivalent in mg/day)				
<5 mg/day	3	7	30.0	*p* = 0.613
5–7.5 mg/day	10	14	41.7	
≥7.5 mg/day or more	6	5	54.5	
History of fracture				
yes	3	3	50.0	*p* = 0.686
no or unknown	16	23	41.0	
Total	19	26	42.2	

GC: glucocorticoid, PSL: prednisolone.

## Data Availability

The datasets used and/or analyzed during the current study are available from the corresponding author on reasonable request.
